# The Role of Sirtuin 1 in Palmitic Acid-Induced Endoplasmic Reticulum Stress in Cardiac Myoblasts

**DOI:** 10.3390/life12020182

**Published:** 2022-01-26

**Authors:** Hsiang-Yu Yang, Jhao-Ying Chen, Yen-Nien Huo, Pei-Ling Yu, Pei-Zhen Lin, Shih-Che Hsu, Shih-Ming Huang, Chien-Sung Tsai, Chih-Yuan Lin

**Affiliations:** 1 Division of Cardiovascular Surgery, Department of Surgery, Tri-Service General Hospital, National Defense Medical Center, Taipei 114, Taiwan; alfie0314@mail.ndmctsgh.edu.tw; 2 Graduate Institute of Life Sciences, National Defense Medical Center, Taipei 114, Taiwan; 3 Department of Biochemistry, National Defense Medical Center, Taipei 114, Taiwan; illy49947125@gmail.com (J.-Y.C.); linda81510@gmail.com (P.-L.Y.); xpeachcj8a8@gmail.com (P.-Z.L.); shihming7102@gmail.com (S.-M.H.); 4 Graduate Institute of Medical Sciences, College of Medicine, Taipei Medical University, Taipei 114, Taiwan; poooooh_1988@hotmail.com; 5 CVie Therapeutics Ltd., Taipei 114, Taiwan; evanhsu6938@gmail.com; 6 Department and Graduate Institute of Pharmacology, National Defense Medical Center, Taipei 114, Taiwan

**Keywords:** palmitate, endoplasmic reticulum stress, sirtuin 1

## Abstract

Background: Lipotoxicity causes endoplasmic reticulum (ER) stress, leading to cell apoptosis. Sirtuin 1 (Sirt1) regulates gene transcription and cellular metabolism. In this study, we investigated the role of Sirt1 in palmitate-induced ER stress. Methods: Both H9c2 myoblasts and heart-specific *Sirt1* knockout mice fed a palmitate-enriched high-fat diet were used. Results: The high-fat diet induced C/EBP homologous protein (CHOP) and activating transcription factor 4 (ATF4) expression in both Sirt1 knockout mice and controls. The Sirt1 knockout mice showed higher CHOP and ATF4 expression compared to those in the control. Palmitic acid (PA) induced ATF4 and CHOP expression in H9c2 cells. PA-treated H9c2 cells showed decreased cytosolic NAD^+^/NADH alongside reduced Sirt1′s activity. The H9c2 cells showed increased ATF4 and CHOP expression when transfected with plasmid encoding dominant negative mutant Sirt1. Sirt1 activator SRT1720 did not affect CHOP and ATF4 expression. Although SRT1720 enhanced the nuclear translocation of ATF4, the extent of the binding of ATF4 to the *CHOP* promoter did not increase in PA treated-H9c2 cells. Conclusion: PA-induced ER stress is mediated through the upregulation of ATF4 and CHOP. Cytosolic NAD^+^ concentration is diminished by PA-induced ER stress, leading to decreased Sirt1 activity. The Sirt1 activator SRT1720 promotes the nuclear translocation of ATF4 in PA-treated H9c2 cells.

## 1. Introduction

Cardiac lipotoxicity, featuring toxic lipid accumulation in the heart, plays a pathological role in the development of obesity induced cardiovascular diseases [1]. The effect of obesity epidemic and dietary fat intake on the development of cardiovascular disease and progression of heart failure has been receiving greater attention in recent years [2,3]. Saturated fatty acids, especially 16-C palmitate, are reported to be more lipotoxic than unsaturated fatty acids in cardiomyocytes [4]. A major mechanism underlying the palmitate-induced cardiomyocyte dysfunction is endoplasmic reticulum (ER) stress, which may promote cell death [5].

ER stress results from an excessive accumulation of misfolded proteins within the ER and triggers a compensatory mechanism, the unfolded protein response (UPR), intended to modulate ER stress and restore ER homeostasis [6]. ER transmembrane proteins, including PKR-like ER kinase (PERK), activating transcription factor 6 (ATF6), and inositol-requiring enzyme 1 (IRE1), are responsible for UPR initiation. The UPR may switch to a proapoptotic signaling pathway to terminate the cell dysfunction. In the apoptosis pathways, activating transcription factor 4 (ATF4) plays a crucial role because it drives the transcription of several apoptosis genes, including a proapoptotic one, C/EBP homologous protein (*CHOP*), also known as DNA damage–inducible transcript 3 protein.

Sirtuin 1 (Sirt1) is a member of the sirtuin family and is an NAD^+^-dependent enzyme performing the deacetylation of target substrates by hydrolyzing NAD^+^ to produce a deacetylated substrate, acetyl-ADP-ribose, and nicotinamide. Furthermore, Sirt1 is capable of deacetylating acetyl-lysine in histones, such as H3K9Ac, for gene transcription modulation. As is known, Sirt1 plays an important part in the regulation of the cellular metabolism, inflammation, the cell cycle, and DNA repair [7]. An impairment of cardiac Sirt1 signaling is reported to contribute to the pathogenesis of cardiovascular diseases [8,9,10]. Additionally, Sirt1 has been shown to provide cardio protection against ER stress–induced cell death through eIF2α deacetylation in a Sirt1 knockout mouse model [11]. However, the role of Sirt1 in saturated fatty acid, such as palmitate, induced ER stress is still not fully understood [12].

The aim of the present study was to investigate the mechanism of palmitate-induced ER stress in cardiomyocytes and to determine the role of Sirt1. We hypothesized that Sirt1 provides protection against palmitate and a high fat diet induces ER stress in cardiac myocytes. Here, we used H9c2 myoblasts and mice with a heart-specific *Sirt1* exon-4 knockout fed a palmitate-enriched high-fat diet (HFD) to identify the signaling pathway involved in palmitic acid (PA)-induced ER stress. We tested whether Sirt1 expression is affected by PA-induced ER stress and explored the possible protective effects of Sirt1. We hope that our current findings provide a novel insight into PA-induced ER stress and into the function of Sirt1 in cardiomyocytes.

## 2. Methods

### 2.1. Genetically Modified and HFD Mouse Models

Animal experiments were all conducted with the approval of the Institutional Animal Care and Use Committee (IACUC, permit No. 19-364) of the National Defense Medical Center (Taipei, Taiwan) and in accordance with the National Institutes of Health guidelines, “Guide for the Care and Use of Laboratory Animals”, on manipulations with experimental animals. The study was carried out in compliance with the ARRIVE guidelines.

The mice with the heart-specific *Sirt1* exon-4 knockout (Sirt1^−/−^) were created by crossing Sirt1^flox/flox^ mice (controls purchased from Jackson Laboratory) with mice carrying α-MHC (myosin heavy chain) promoter–driven *Cre* in a C57BL/6J background (α-MHC-Cre mice, courtesy of Prof. M. Schneider, Imperial College London) and are currently in use in the laboratory [13]. Six-week-old mice were separately fed either a standard diet (SD) (10% kcal fat, D17071303i, Research Diets, New Brunswick, NJ, USA) or a palmitate-enriched HFD (60% kcal fat, D16042106i, Research Diets, New Brunswick, NJ, USA) for 8 weeks and then were euthanized to collect the hearts for subsequent experiments. The animals were kept at a temperature of 21 ± 1 °C on a controlled 12:12 h light-dark cycle with *ad libitum* access to deionized drinking water before the experiments.

### 2.2. Cardiomyocyte Isolation

Ventricular myocytes were enzymatically dissociated as previously described [14], with modifications. Briefly, mice were euthanized using a mixture of Zoletil 50 and xylazine, and the hearts were excised and cannulated via the aorta to a Langendorff perfusion system at 37 °C. Each heart was first perfused with normal Tyrode’s solution (137 mM NaCl, 1.8 mM CaCl_2_, 0.5 mM MgCl_2_, 5.4 mM KCl, 10 mM glucose, and 10 mM HEPES [pH adjusted to 7.4 with NaOH]) for 10 min and digested with a Ca^2+^-free solution (120 mM NaCl, 5.4 mM KCl, 1.2 mM MgSO_4_, 1.2 mM KH_2_PO_4_, 6 mM HEPES, 10 mM glucose, and 10 mM taurine [pH adjusted to 7.4 using NaOH]) containing 1 mg/mL collagenase (Type I, Sigma-Aldrich, St. Louis, MO, USA) and 0.06 mg/mL proteinase (type XIV, Sigma-Aldrich, St. Louis, MO, USA). After the perfusion, the heart was disconnected from the cannula, cut into small pieces, and gently triturated with a plastic transfer pipette, and the homogenate was filtered through a nylon mesh. The dissociated cells were stored in normal Tyrode’s solution at 20–22 °C. Rod-shaped cells with clear striations and no granulation were used within 6–8 h for all the experiments.

### 2.3. H9c2 Culture and Plasmid Transfection

The H9c2 rat myoblast cell line (BCRC60096) was purchased from the Bioresource Collection and Research Center of the Food Industry Research and Development Institute (Taipei, Taiwan). The cells were cultured in Dulbecco’s modified Eagle’s medium (DMEM) supplemented with 10% of fetal bovine serum, 150 U/mL penicillin, and 150 mg/mL streptomycin. The cells were incubated at 37 °C in 5% CO_2_/95% air. Confluent cells were detached with a 0.05% trypsin/0.02% EDTA solution and subcultured in 6-well culture plates to obtain the second passage as previously described [15]. To check the protein levels and mRNA expression of CHOP and ATF4 induced by PA, the H9c2 cells were treated with PA with concentrations ranging from 0 to 200 μM for 24 h. cDNA sequences of full-length mutant Sirt1 (H363Y) were obtained from Addgene (Cambridge, MA, USA) [16,17]. cDNAs of mutant Sirt1 (H363Y) were cloned into the pSG5.HA vector [18]. On the following day, the cells were plasmid-transfected by means of the Lipofectamine 3000 Reagent (Thermo Fisher Scientific Co., Carlsbad, CA, USA) according to the manufacturer’s instructions. After 24 h transfection, the H9c2 cells were treated with 150 μM PA for 12 h.

### 2.4. Immunoblotting Analysis

Ventricular tissues were homogenized in FastPrep-24 5G (MP Biomedicals, Irvine, CA, USA) with RIPA buffer (100 mmol/L Tris-HCl pH 8.0, 0.1% of sodium dodecyl sulfate, 1% of Triton X-100, and 150 mmol/L NaCl) containing a protease inhibitor cocktail (Roche, Basel, Switzerland), followed by centrifugation at 15,000 rpm for 15 min at 4 °C. The protein content was determined in the supernatants, according to the DC Protein Assay instruction manual (Bio-Rad, Hercules, CA, USA). The H9c2 cells were lysed in RIPA buffer (100 mM Tris-HCl pH 8.0, 150 mM NaCl, 0.1% of SDS, and 1% of Triton X-100) and then centrifuged for 15 min at 15,000 rpm at 4 °C. The protein extracts from heart tissue and from cultured H9c2 cells were separated by SDS polyacrylamide gel electrophoresis and then transferred to polyvinylidene difluoride membranes (Merck Millipore, Burlington, MA, USA), which were incubated with the following antibodies: anti-α-Tubulin (1:10,000, Proteintech, Manchester, UK), anti-GAPDH (1:10,000, Proteintech, UK), anti-CHOP (1:800, Cell Signaling Technology, Danvers, MA, USA), anti-ATF4 (1:800, Santa Cruz Biotechnology, Dallas, TX, USA), anti-Sirt1 (1:800, Merck Millipore, Burlington, MA, USA), and anti-ac-H3K9 (1:1000, Merck, Kenilworth, NJ, USA). Subsequently, the membranes were incubated with anti-mouse (Santa Cruz Biotechnology, Dallas, TX, USA, sc-2056) or anti-rabbit (Santa Cruz Biotechnology, Dallas, TX, USA, sc-2004) secondary IgG antibodies at a dilution of 1:10,000. Immunoreactive proteins were detected via enhanced chemiluminescence (GE Healthcare, Chicago, IL, USA) and were then quantified in the ImageJ software 1.5 (NIH, Bethesda, MD, USA).

### 2.5. RT-PCR Analysis

Total RNA was extracted from heart tissue and cultured H9c2 cells using the Total RNA reagent (Bioman, New Taipei City, Taiwan). Next, 1 μg of total RNA was reverse-transcribed using the Moloney murine leukemia virus (MMLV) reverse transcriptase (Epicentre Biotechnologies, Madison, WI, USA) as per the manufacturer’s instructions. The resultant cDNA was quantified by quantitative RT-PCR in an Illumina ECO™ Real-Time PCR system. Cycle threshold (C_t_) values for target mRNAs were normalized to the housekeeping gene *GAPDH*, and the relative gene expression was calculated by the 2^−ΔΔCt^ method.

Primer sequences were as follows: *GAPDH* forward 5′-GGATACTGAGAGCAAGAGAGAGG-3′ and reverse 5′-TCCTGTTGTTATGGGGTCTGG-3′, *CHOP* forward 5′-CCAGCAGAGGTCACAAGCAC-3′ and reverse 5′-CGCACTGACCACTCTGTTTC-3′, and *ATF4* forward 5′-CCTGACTCTGCTGCTTATATTACTCTAAC-3′ and reverse 5′-ACTCCAGGTGGGTCATAAGGTTTG-3′.

### 2.6. Cellular NAD^+^(H) Levels, and Cytoplasmic and Nuclear Extract Preparation

The cytosolic NAD^+^/NADH ratio was determined using the NAD^+^/NADH Quantitation Colorimetric Kit (BioVision K337-100) as per the manufacturer’s instructions. The H9c2 cells were washed twice in ice-cold PBS and detached with PBS. After the removal of the supernatant, cytoplasmic and nuclear proteins were extracted from the cells by means of the Cytoplasmic and Nuclear Protein Extraction Kit (BIOTOOLS Co., Ltd., New Taipei City, Taiwan), according to the manufacturer’s instructions.

### 2.7. A Chromatin Immunoprecipitation (ChIP) Assay

ChIP was performed with the SimpleChIP^®^ Enzymatic Chromatin IP Kit (Cell Signaling Technology, Danvers, MA, USA). Briefly, cells were incubated with 1% formaldehyde at room temperature for 10 min for cross-linking proteins to DNA. Chromatin was sonicated, of which 10 mg was subjected to immunoprecipitation with antibodies [anti-ATF4 (Santa Cruz Biotechnology, Dallas, TX, USA), anti-ac-H3K9 (Merck, Kenilworth, NJ, USA), or negative control (Normal Mouse IgG; Cell Signaling Technology, Danvers, MA, USA)] at 4 °C overnight with rotation. The following day, chromatin/antibody complexes were pulled down from the solution by incubation with salmon sperm DNA–saturated 50% protein A/G–Sepharose beads at 4 °C for 2 h. The cross-linking was reversed by heating at 65 °C for 30 min, followed by treatment with 100 g/L proteinase K at 65 °C for 2 h. DNA purification was performed using the kit mentioned above, and the purified DNA was analyzed by PCR. The primers for *CHOP* were forward 5′-AAGTTCAGGAAGGACAGCCG-3′ and reverse 5′-CGTTATCTCGGACCCGGAAG-3′.

### 2.8. Acquisition Systems and Statistical Analysis

Continuous variables were expressed as the mean ± standard error of the mean. A Student’s *t*-test or Pearson’s Chi-square test was performed to evaluate the differences. GraphPad Prism 5 (Systat Software 3.5, Inc., San Jose, CA, USA) was used for statistical comparisons. In figures, ”n” stands for the total number of cells per heart (n = cells/hearts), and ”N” is the number of mice. Statistical significance is indicated by *, **, and *** for *p* < 0.05, *p* < 0.01, and *p <* 0.001, respectively.

## 3. Results

### 3.1. The Induction of CHOP and ATF4 Expression in Cardiomyocytes of Mice and H9c2 Cells by HFD and PA, Respectively

**Figure 1:**
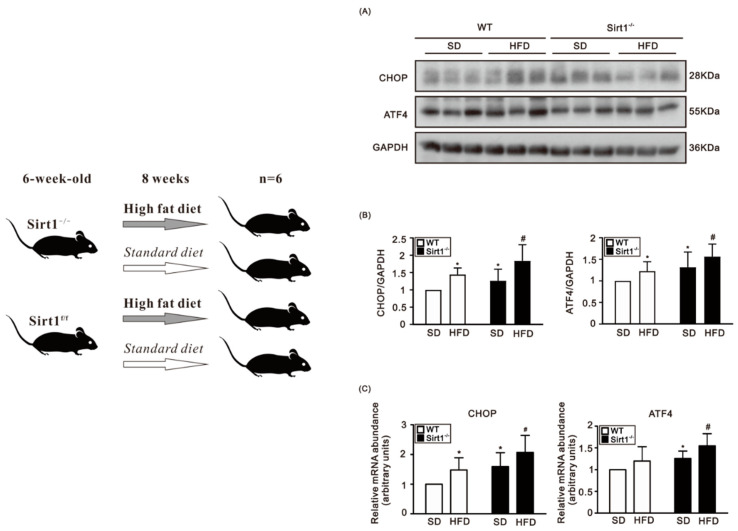
(**Left**) Scheme of experimental animal usage. (**Right**) Protein levels and mRNA expression of CHOP and ATF4 in ventricular tissues from control and Sirt1^−/−^ mice fed either the SD or HFD. (**A**) A representative immunoblots of CHOP and ATF4 in control and Sirt1^−/−^ mice fed with either the SD or HFD. (**B**) The HFD increased protein levels of CHOP and ATF4 in WT mice as compared to those fed with the SD. The protein levels of CHOP and ATF4 were higher in Sirt1^−/−^ mice compared to the control mice fed either the SD or HFD (N = 6 for each group; * compared to WT mice on the SD, # compared to WT mice on the HFD; * *p* < 0.05; # *p* < 0.05). (**C**) Relative mRNA expression of CHOP and ATF4 was higher in Sirt1^−/−^ mice compared to the control mice fed with either the SD or HFD (N = 6 for each group; * compared to WT mice on the SD, # compared to WT mice on the HFD; * *p* < 0.05; # *p* < 0.05). (SD: standard diet; HFD: high fat diet; WT: wide type).

**Figure 2:**
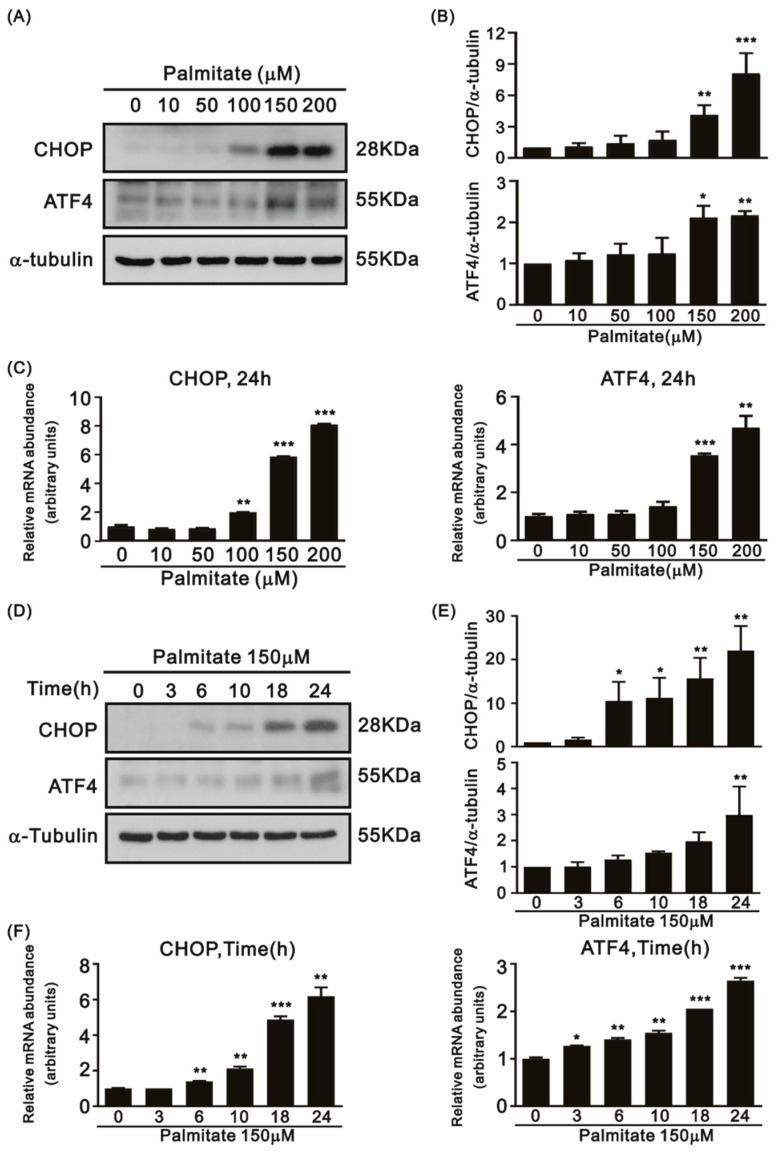
Protein levels and mRNA expression of CHOP and ATF4 in the H9c2 cells treated with PA. (**A**) A representative immunoblot and (**B**) mean data for CHOP and ATF4 in the H9c2 cells treated with PA (0–200 μM) at 24 h (n = 3; asterisk (s): compared to the 0 μM PA control group; * *p* < 0.05, ** *p* < 0.01, *** *p* < 0.001). (**C**) Relative levels of *CHOP* and *ATF4* mRNAs in H9c2 cells after PA (0–200 μM) treatment at 24 h (n = 3; asterisk (s): compared to the 0 μM PA control group; ** *p* < 0.01, *** *p* < 0.001). (**D**) A representative immunoblot and (**E**) mean data for CHOP and ATF4 in H9c2 cells treated with PA (150 μM, 0–24 h) (n = 3; asterisk (s): compared to the 0 μM PA control group; * *p* < 0.05, ** *p* < 0.01). (**F**) Relative expression levels of *CHOP* and *ATF4* mRNA in H9c2 cells after PA (150 μM, 0–24 h) treatment (n = 2; asterisk (s): compared to the 0 μM PA control group; * *p* < 0.05, ** *p* < 0.01, *** *p* < 0.001).

The HFD is one of the inducers of ER stress [19,20]. To address the role of Sirt1 in HFD-induced ER stress in cardiomyocytes, we fed 8-week-old Sirt1^f/f^ and Sirt1^−/−^ mice with HFD for 8 weeks. CHOP and ATF4 protein levels and mRNA expression were higher in the control mice fed with the HFD compared to those fed the SD (Figure 1A–C). Sirt1^−/−^ mice showed increased protein levels and mRNA expression of CHOP and ATF4 as compared to the control mice fed either the SD or HFD (Figure 1B,C). We exposed H9c2 cells to PA and investigated the expression of CHOP and ATF4. The protein levels and mRNA expression of both ATF4 and CHOP in H9c2 cells were significantly increased in the PA treatment in a dose-dependent manner (Figure 2A–C) and in a time-dependent manner (Figure 2D–F).

### 3.2. Palmitate Decreases Sirt1′s Deacetylating Activity Mediated by the Downregulation of Cytosolic NAD^+^ in H9c2 Cells

**Figure 3:**
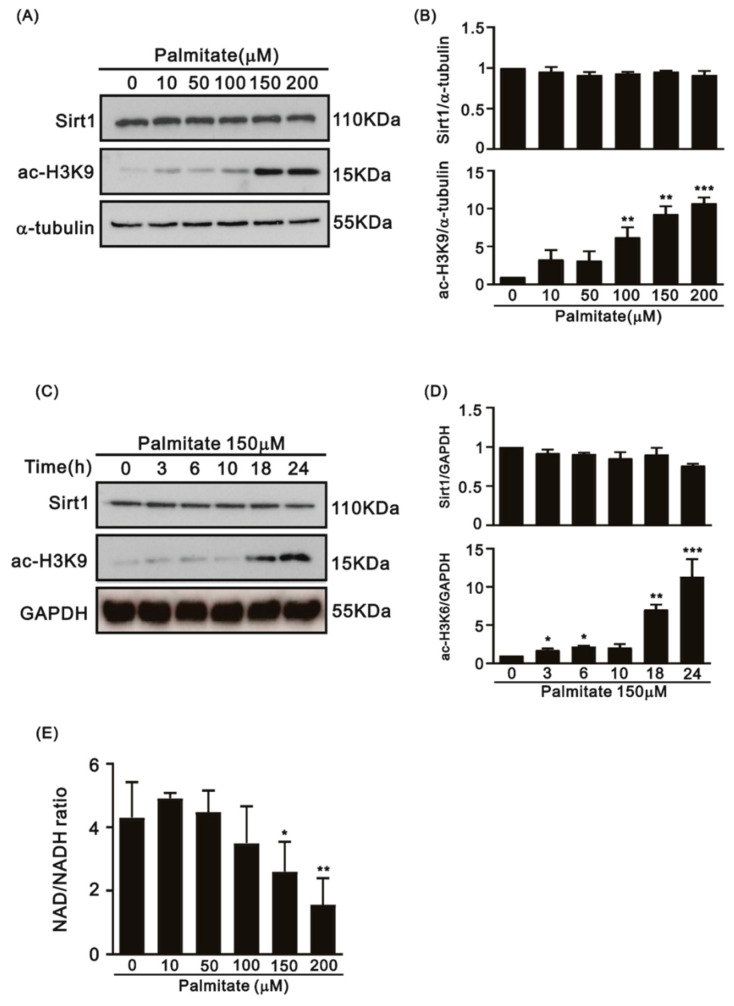
Protein levels of Sirt1, the amount of H3K9Ac, and the cytosolic NAD^+^/NADH ratio in H9c2 cells treated with PA. (**A**) A representative immunoblot and (**B**) mean data for Sirt1 and H3K9Ac in H9c2 cells treated with PA (0–200 μM) at 24 h (n = 3; asterisk: compared to the 0 μM PA control group; ** *p* < 0.01, *** *p* < 0.001). (**C**) A representative immunoblot and (**D**) mean data for Sirt1 and H3K9Ac in H9c2 cells treated with PA (150 μM, 0–24 h) (n = 3; asterisk (s): compared to the 0 μM PA control group; * *p* < 0.05, ** *p* < 0.01, *** *p* < 0.001). (**E**) The cytosolic NAD^+^/NADH ratio in the H9c2 cells treated with PA decreased in a PA dose-dependent manner (n = 3; * *p* < 0.05, ** *p* < 0.01).

As is known, Sirt1 deacetylates acetyl-lysine in histones, e.g., H3K9Ac, to modulate gene transcription [21]. To investigate the function of Sirt1 under PA-induced ER stress in H9c2 cells, we quantified Sirt1 protein expression and acetyl-lysine9 in histone H3 (H3K9Ac), a primary target of Sirt1. While the protein level of Sirt1 remained unchanged, the level of H3K9Ac significantly increased in a dose-dependent and time-dependent manner in H9c2 cells after treatment with PA (see Figure 3A,B for the dose-course experiment and Figure 3C,D for the time-course experiment). The increased amount of H3K9Ac indicated the decreased enzymatic activity of Sirt1 may be not mediated by the downregulation of the Sirt1 protein amount. We next examined whether the cellular NAD^+^/NADH ratio was affected, which may be responsible for the reduced deacetylating activity of Sirt1, under PA-induced ER stress. Our data showed that NAD^+^/NADH ratios significantly decreased in H9c2 cells treated with PA in a dose-dependent manner (Figure 3E).

### 3.3. Sirt1 Reduces mRNA and Protein Expression of ATF4 and CHOP in H9c2 Cells

**Figure 4:**
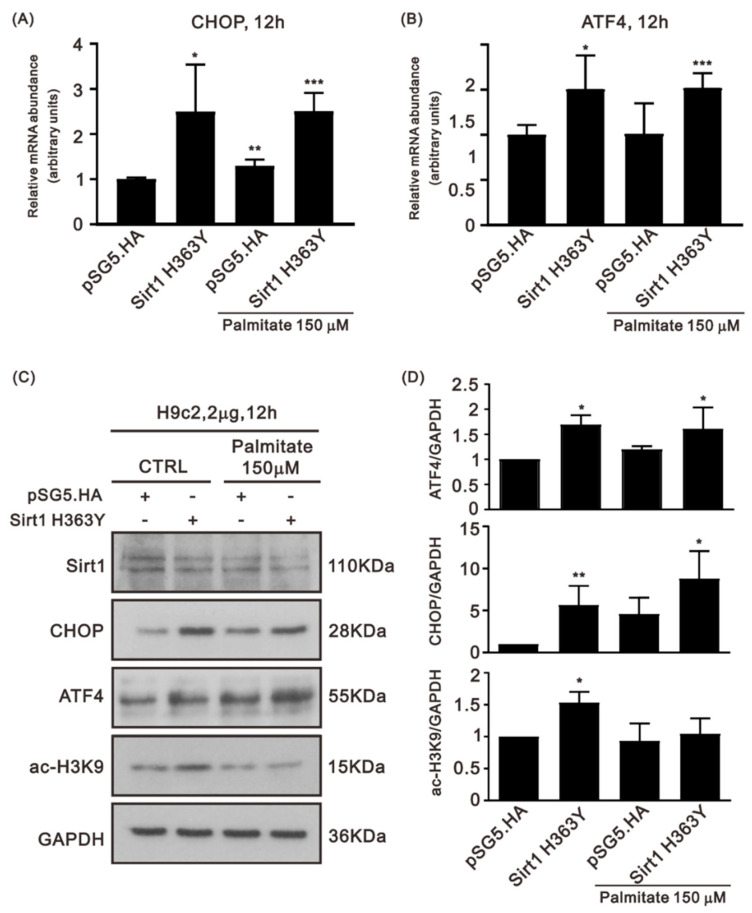
Effects of transfection with the plasmid encoding the Sirt1 H363Y mutant in H9c2 cells. (**A**,**B**) Relative mRNA expression of CHOP and ATF4 in pSG5.HA- or H363Y mutant Sirt1 plasmid–transfected H9c2 cells treated or not treated with PA (n = 3; asterisk (s): compared to the pSG5.HA group; * *p* < 0.05, ** *p* < 0.01, and *** *p* < 0.001). (**C**,**D**) The protein expression of CHOP and ATF4 in pSG5.HA- or H363Y mutant Sirt1 plasmid–transfected H9c2 cells treated or not treated with PA (n = 3; asterisk (s): compared to the pSG5.HA group; * *p* < 0.05 and ** *p* < 0.01).

It is important to verify the decreased ratio of NAD^+^ to NADH to assess Sirt1′s deacetylating enzymatic activity. One dominant negative Sirt1 mutant (H363Y) has been found to lose its deacetylating activity [22]. Hence, we suppressed the enzymatic activity of Sirt1 via this dominant negative H363Y mutation in H9c2 cells to determine whether the abrogation of Sirt1 enzymatic activity affects the expression of CHOP and ATF4. Sirt1 H363Y induced mRNA and protein expression of CHOP and ATF4 in H9c2 cells (Figure 4A,B). After PA treatment, the protein expression of CHOP and ATF4 increased in Sirt1 H363Y–transfected H9c2 cells (Figure 4C,D). On the contrary, H3K9Ac upregulation by Sirt1 H363Y was attenuated by the PA treatment.

### 3.4. SRT1720 Enhances Nuclear Translocation of ATF4 and Reduces Histone H3K9 Acetylation in the CHOP Promoter

**Figure 5:**
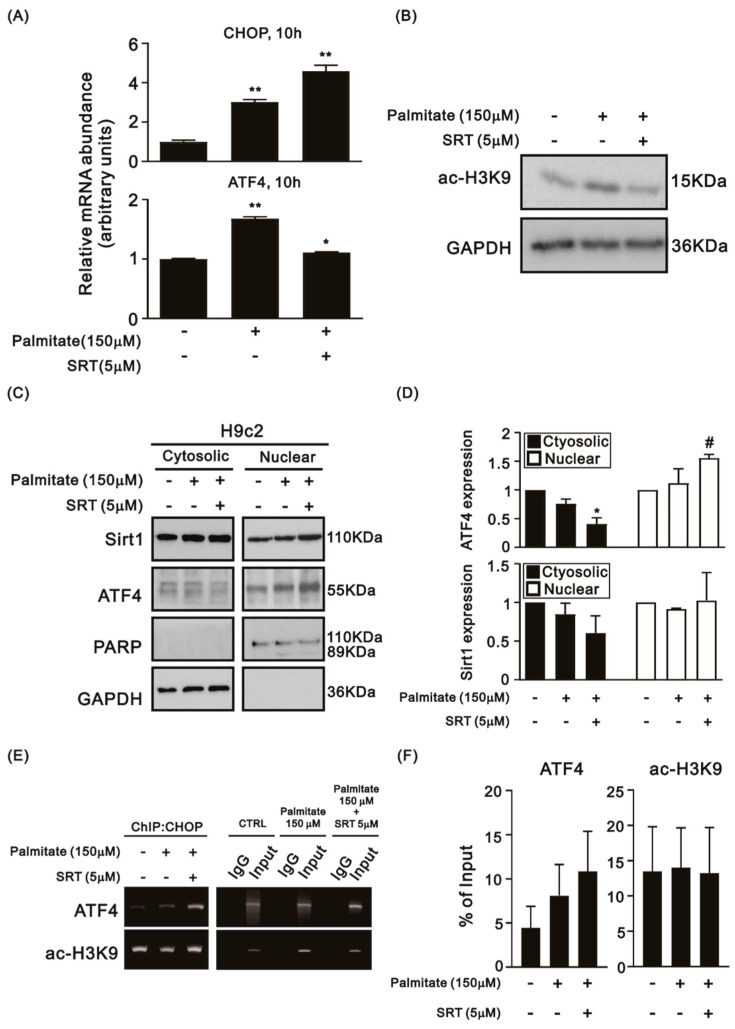
Effects of SRT1720 on CHOP and ATF4 in the H9c2 cells treated with PA. (**A**) The relative mRNA expression of CHOP in PA-treated H9c2 cells with or without SRT1720 (n = 3; * *p* < 0.05, ** *p* < 0.01). (**B**) A representative immunoblots of ATF4 and H3K9Ac in PA-treated H9c2 cells with or without SRT1720 (n = 3). (**C**,**D**) The expression of ATF4 and Sirt1 in cytosolic and nuclear fractions (n = 3; * compared to the cytosol control; # compared to the nuclear control; * *p* < 0.05, # *p* < 0.05). (**E**,**F**) ChIP for assessing the interaction between ATF4 and the *CHOP* promotor and between H3K9Ac and the *CHOP* promotor in PA-treated H9c2 cells with or without SRT1720 (n = 3).

The above-mentioned data indicated that PA reduces the cytosolic NAD^+^ amount, thereby suppressing the enzymatic activity of Sirt1 in H9c2 cells. Accordingly, we applied a Sirt1 activator, SRT1720, to address the role of the deacetylating activity of Sirt1 in the regulation of ATF4 and the CHOP expression in H9c2 cells. Furthermore, SRT1720 did not suppress mRNA expression of CHOP and ATF4 in PA-treated H9c2 cells (Figure 5A). SRT1720 reduced the levels of H3K9Ac in PA-treated H9c2 cells, suggesting that it is an activator of Sirt1′s deacetylating activity (Figure 5B). Under ER stress, ATF4 may migrate to the nucleus, bind to target genes, and regulate their transcription [23]. Next, we tested whether SRT1720 affects the cytosolic and nuclear fractions of Sirt1 and ATF4 in H9c2 cells with or without PA. Nuclear translocation of ATF4 in H9c2 cells was increased by the PA treatment and further strengthened when combined with SRT1720 treatment (Figure 5C,D). Neither PA nor SRT1720 influenced the cytosolic and nuclear fractions of Sirt1 (Figure 5C,D). The ChIP results showed that in H9c2 cells, PA did not affect the recruitment of ATF4 to the *CHOP* promoter (Figure 5E,F). SRT1720 did not have effect on the amount of H3K9Ac in the *CHOP* promoter in PA-treated H9c2 cells (Figure 5E,F).

## 4. Discussion

Lipotoxicity is an important contributor to cardiac dysfunction in obesity associated heart disease [24,25]. An excessive uptake of fatty acids can result in either enhanced oxidation or abnormal accumulation of toxic lipid species such as ceramides and diacylglycerides, causing lipotoxicity in the heart and other solid organs, and the one of crucial underlying mechanism is ER stress and impaired UPR signaling [26]. Some evidence indicates that the activation of Sirt1/AMPK signaling may prevent cells from fatty acid–induced oxidative stress and inflammation [27], but the participation of Sirt1 in lipotoxicity-induced ER stress remains unclear. In the present study, we utilized mice fed a palmitate-enriched HFD and palmitate-treated H9c2 cells as in vivo and in vitro models of lipotoxicity and examined the cellular consequences, including ER stress and Sirt1 activity, as well as the crosstalk between these cellular phenomena.

There are studies showing that the lipotoxicity associated with ER stress increases *ATF4* and *CHOP* mRNA expression, but the underlying mechanism still needs to be clarified [26,28,29]. Our data reveal that palmitate induces the expression of ER stress markers CHOP and ATF4 in vivo and in vitro, in line with other reports [26,28,29]. One study suggested that palmitate-induced cardiomyocyte dysfunction is mediated by ER stress and thereby promotes cell death [5]. Furthermore, Sirt1 may confer cardio-protection against ER stress. Alexandre et al. have reported that cardiac Sirt1 deficiency increases the contractile dysfunction caused by ER stress in a Sirt1 knockout mouse model, and the mechanism may involve eIF2α deacetylation [11]. According to our results, Sirt1^−/−^ cardiomyocytes show higher expression of CHOP and ATF4 as compared to cardiomyocytes from the control mice fed either the SD or HFD, also suggesting a protective role of Sirt1 against HFD-induced ER stress in cardiomyocytes.

As an NAD^+^-dependent reaction, the protein deacetylation catalyzed by Sirt1 is accompanied by the hydrolysis of NAD^+^. Therefore, we propose that the decreased Sirt1 activity is associated with reduced NAD^+^ concentration in palmitate-treated H9c2 cells, in line with other reports [30,31]. Some research has revealed that changes in cytosolic NAD^+^ levels alter Sirt1 activity [12,32,33]. Additionally, one report has shown that a reduced cellular NAD^+^ concentration, resulting from the conversion of NAD^+^ to NADH by the glucose metabolic pathway, leads to lower Sirt1 activity [34]. As a consequence of its dependence on NAD^+^ and therefore on the cellular NAD^+^/NADH ratio, Sirt1 has emerged as a key metabolic sensor with respect to various tissues [35]. There is evidence [36,37] that in a high-energy state, such as that associated with an HFD and obesity, Sirt1 activity may decline with a decreased level of NAD^+^. Therefore, we suggest that palmitate-induced ER stress diminishes the cytosolic level of NAD^+^, which in turn reduces Sirt1 enzymatic activity.

Our results indicate that the dominant negative Sirt1 H363Y mutant increases the expression of ATF4 and CHOP, also implying a protective role of Sirt1 (via its enzymatic activity) against ER stress [11]. Nevertheless, PA did not raise either ATF4 or CHOP expression in the H9c2 cells transfected with the plasmid encoding the Sirt1 H363Y mutant, and the same was true for H3K9Ac. We believe that the plasmid transfection of H9c2 cells attenuates the influence of PA or PA weakens the effects of the plasmid transfection. In addition, our data indicate that autophagy-related proteins beclin 1 and p63 are upregulated in the H9c2 cells treated with PA (Supplemental Figure S1). PA-induced autophagy has been shown to abrogate the partial apoptosis caused by PA [38]. Therefore, ATF4 or CHOP expression in the H9c2 cells transfected with the Sirt1 H363Y mutant may be abrogated by the autophagy induced by PA. The increase in the H3K9Ac level in PA-treated H9c2 cells was attenuated by SRT1720 treatment, implying an increased deacetylation function of Sirt1, although the NAD^+^ concentration was not restored under these conditions. On the other hand, the PA-induced expression of ATF4 was not weakened by SRT1720 treatment. Furthermore, our findings show that the translocation of ATF4 to the nucleus increases upon SRT1720 treatment; therefore, the increased nuclear recruitment may enhance the gene regulatory function of ATF4. The amount of binding between ATF4 and the *CHOP* promoter did not affect with PA treatment. With PA plus SRT1720 treatment, the extent of binding between ATF4 and the *CHOP* promoter did not increase, although SRT1720 led to more ATF4 becoming available in the nucleus. Additionally, SRT1720 strengthened the activity of Sirt1 but only had a limited impact on the CHOP and ATF4 expression induced by PA treatment, suggesting the involvement of more than a deacetylating activity. The protein–protein interaction and nuclear localization are being researched in more detail to decipher the current complicated findings.

As a possible metabolic sensor, the activity of Sirt1 and the level of NAD^+^ could be used for assessment of ER stress. Therefore, restoring the activity of Sirt1 and the level of NAD^+^ could provide clinically therapeutic potential for protecting various organs such as the heart from ER stress-induced injury [39]. These findings may also provide potential clinical application for Sirt1 in the treatment of metabolic syndrome associated cardiovascular disease. Future studies on Sirt1 overexpression using plasmid transfection could be conducted to examine if increased amount of Sirt1 provides protection against PA-induced ER stress via post-translational modification of target substrates. Whether Sir1 has protective role in PA-induced ER stress associated endothelial dysfunction may be another future direction to be investigated.

In conclusion, PA-induced ER stress is mediated through the upregulation of ATF4 and CHOP mRNAs and proteins. PA reduces the amount of cytosolic NAD^+^, which in turn suppresses Sirt1 activity. Nevertheless, the Sirt1 activator SRT1720 does not attenuate the expression of CHOP and ATF4 induced by PA, but enhances the nuclear translocation of ATF4.

## Data Availability

The data of the present study are available from the corresponding authors upon reasonable request.

## Supplementary Materials

The following supporting information can be downloaded at: https://www.mdpi.com/article/10.3390/life12020182/s1, Figure S1: Protein levels of autophagy-related proteins in the H9c2 cells treated with PA.
