# Supplementary material for: The Role of Sirtuin 1 in Palmitic Acid-Induced Endoplasmic Reticulum Stress in Cardiac Myoblasts
Source: Life (Basel). 2022 Jan 26;12(2):182. doi: 10.3390/life12020182 (PMC8878829; doi:10.3390/life12020182)
Supplement: Supplementary file 1 [file life-12-00182-s001.zip › life-1557249-supplementary.pdf]

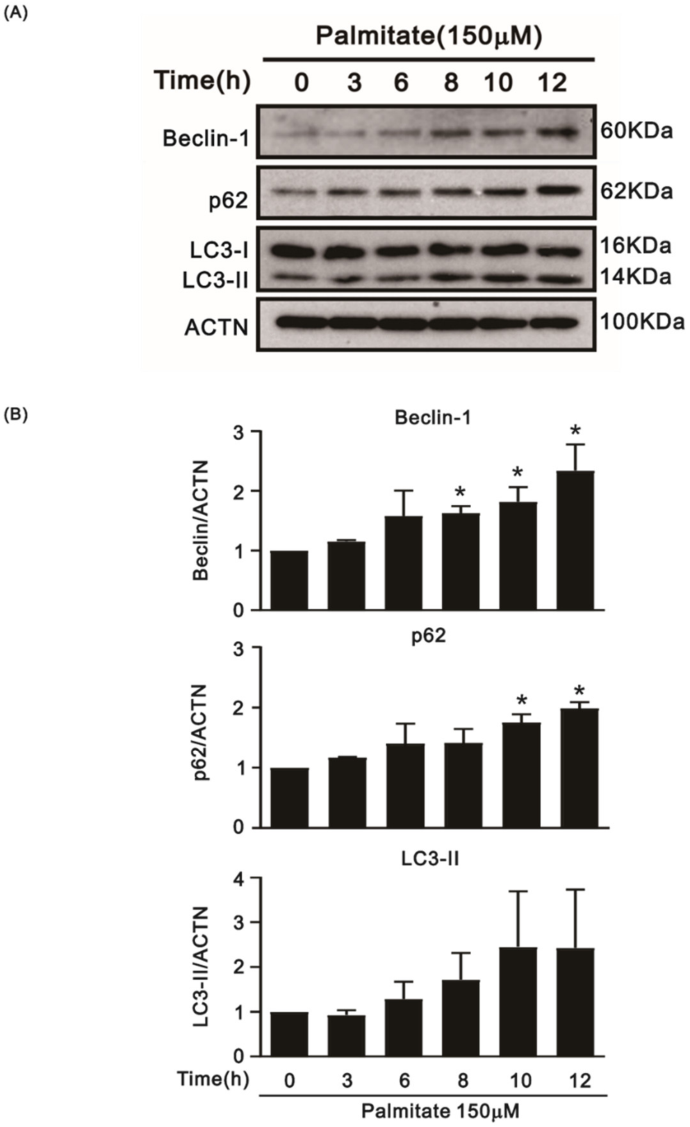

Figure S1. Protein levels of autophagy-related proteins in the H9c2 cells treated with PA.

(**A**) A representative immunoblot of LC3B, beclin 1, and p62 in the H9c2 cells treated with PA. (**B**) Autophagy-related proteins beclin 1 and p62 in H9c2 cells were upregulated by the PA treatment (n = 3; *compared to the 0 h group; *$p < 0.05$).
